# Matching Detection of Crane Hook and Ladle Lug before Ladle Hoisting

**DOI:** 10.3390/s19245389

**Published:** 2019-12-06

**Authors:** Jiashi Lyu, Ruchuan Shi, Yang Yang, Tao Han

**Affiliations:** School of Electronic Information and Electrical Engineering, Shanghai Jiao Tong University, Shanghai 200240, China; lmr101611@sjtu.edu.cn (J.L.); srcno1@sjtu.edu.cn (R.S.); yangyang2015@sjtu.edu.cn (Y.Y.)

**Keywords:** ladle hoisting, surface acoustic wave, SAW RFID localization, geometric mapping, synthetic aperture

## Abstract

Reliable matching between the crane hook and ladle lug is a key requirement for the safe hoisting of a ladle in steelmaking. A novel method is proposed to detect the matching between the hook and lug using surface acoustic wave radio frequency identification (SAW RFID) localization. SAW RFID tags are attached to the surface of the lug and the hook. The position of the lug is estimated via a geometric mapping approach with a special position of the tag and the reader’s antenna, and the position of the hook is estimated by a synthetic aperture approach with the hook’s movement pattern. Afterwards, the matching judgement is determined based on the relative position between the hook and lug. The proposed method employs only two SAW tags and two reader antennas, facilitating installation and routine maintenance. Numerical simulation and physical experiments demonstrate that the proposed method works effectively for matching detection.

## 1. Introduction

**Figure 1:**
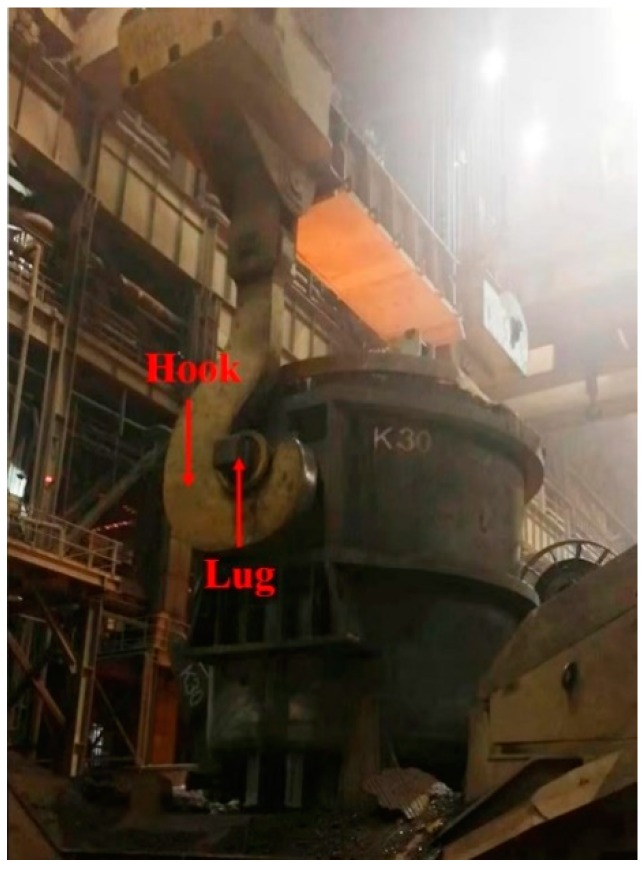
Steel ladle hoisting.

The ladle is the carrier of molten steel, and its transportation is the basic element of steel production. As shown in Figure 1, the lifting of a molten steel ladle mainly relies on the gantry crane in the steelmaking workshop. The hooks of the gantry crane are hung on the lifting lugs on both sides of the ladle to hoist it, and the ladle circulates between the steelmaking links [1]. Before lifting the hook, reliable matching between the hook and the lug should be strictly controlled; otherwise, the huge power of the gantry crane will cause the steel ladle to capsize and potentially elevate the risk of major accidents [2].

Presently, the operator of a gantry crane needs to continuously adjust the position of the hook under auxiliary commands of the ground staff in order to match the hook and lug. However, repeated adjustments under auxiliary commands are fairly inefficient and jeopardize the productivity of the workshop. Moreover, manual observation is subjected to possible misjudgment due to fatigue, movement, and so on, which introduces serious possible security risks. In principle, machine vision can assist or replace manual commands. However, the strong light and complex metal backgrounds in steelmaking workshops make visual imaging and image segmentation extremely difficult. As a result, incorporating machine vision techniques is sometimes impossible. Therefore, reliable matching detection of the hook and lug requires further research.

Reliable matching of the hook and lug can be gauged by their relative positions. The key to actual hoisting is to align the hook’s groove with the lug so that the lug can be hooked accurately when the hook lifts up. One possible method for reliable matching detection is localization of the hook and lug, with which the relative positions of the hook and lug can be obtained. As a result, the problem of reliable matching detection of the lug and hook can be transformed into a problem of localizing the hook and lug.

However, the harsh environment of a steel plant limits the application of existing localization technology. As far as global positioning system (GPS) technology is concerned, the closed environment of a steelmaking workshop means that the signal transmission of GPS is blocked. Laser ranging, coding cables, and other ranging methods have considerable limitations in the target position, thus requiring a complicated layout [3,4], wherein installation or maintenance will slow down, or even stop, steelmaking production. Moreover, the temperature on the ladle’s surface will reach up to 350 °C, which makes the active localization sensors or conventional radio frequency identification (RFID) work unreliably and is unsustainable [5]. In addition, a steelmaking workshop generates strong environmental clutter and multipath fading signals, which restrict the reading distance of the above sensors and RFID [6].

Despite the limited success of the aforementioned technologies, a special RFID based on surface acoustic wave (SAW) delay-line tags offers obvious advantages in the localization of lugs and hooks with its unique mechanism. As the SAW tag is based on piezoelectric materials, it can work reliably in an environment above 300 °C for a long time [7]. Meanwhile, SAW propagation in delay-line SAW tags offers a few microseconds time delay for the tag’s backscattered signal, which separates the transmission and reception of the interrogation signal. Unexpected signals, such as environmental clutter, multipath signals, and leakage signals from the transmission circuits, will be attenuated greatly during the tag’s time delay [8,9].

However, existing SAW RFID localization methods are mainly based on range detection. On the one hand, the ranging errors of these systems only reach tens of centimeters [9,10,11], which hardly meets the requirements for reliable matching detection. On the other hand, to achieve multi-dimensional localization, the number of reader antennas generally exceeds the number of localization dimensions [12,13,14], which makes the antenna installation fairly difficult. This paper proposes a simple and affordable method that requires only two SAW tags and two reader antennas to achieve matching detection of the lug and hook. With prior knowledge of the lug and the hook, the position of the lug is estimated based on geometric mapping, and the position of the hook is determined based on synthetic aperture. Numerical simulations have been performed according to a real scene with phase error and position deviation, which shows the localization error distribution of the proposed method. Furthermore, a testing system with a self-developed SAW RFID has been developed; and this testing system has been used to simulate the real scene to verify the proposed method. Both the numerical simulation and real-world experimental results validate the feasibility of this method.

The remainder of this paper is organized as follows. In Section 2, the requirements of reliable matching detection using SAW RFID localization is analyzed. Section 3 introduces the basis of SAW RFID localization. Section 4 discusses the SAW RFID localization algorithm for matching detection. In Section 5 and Section 6, the simulation and experimental results of the proposed method are discussed. Finally, Section 7 presents the conclusions.

## 2. Matching Detection of the Hook and Lug

**Figure 2:**
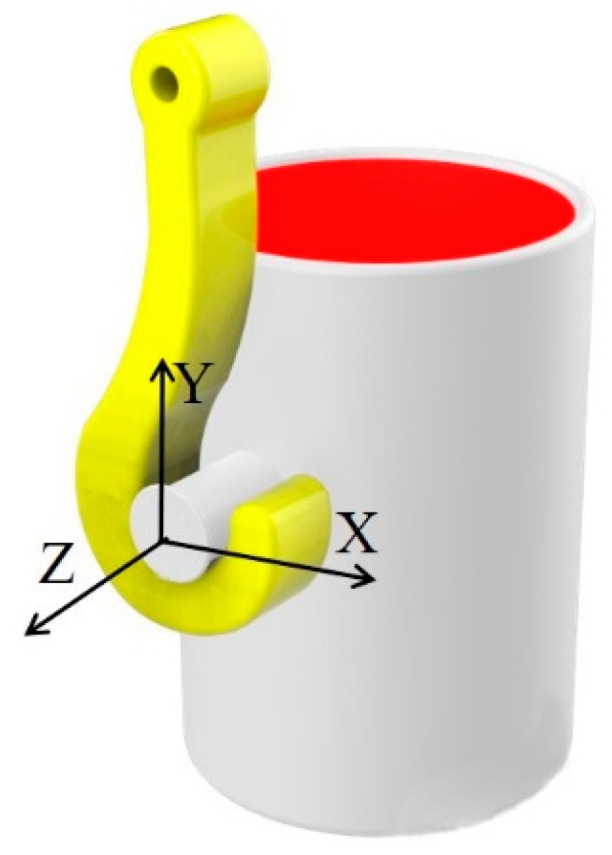
The definition of coordinates.

For convenience of description, the coordinate system, as shown in Figure 2, has been established. The direction parallel to the ground and the surface of the lug is denoted as X. The direction perpendicular to the ground and the surface of the lifting lug is denoted as Y. Finally, the direction perpendicular to the surface of the lug is denoted as Z. The space axes system here obeys the right-hand rule.

After the lug and the hook have been correctly matched in one hoisting, a fixed point is selected in the workshop as the origin of the coordinate system $S_{0}=(0,0,0$) without a loss of generality.

### 2.1. Reliable Matching Analysis

Due to the ladle’s circulation, the position of the ladle and the trajectory of the gantry crane vary in three-dimensional space under multiple hoisting. These position deviations make it difficult to align the lug and the hook groove. This section analyzes the conditions for the reliable matching of the lug of the hook within the space scope.

The distance between the gantry crane’s two hooks along the direction of the *z*-axis is fixed and slightly larger than that of the two lugs of the ladle. Owing to the limitations of this mechanical structure, the lug and the hook on each side are always confined to an XOY plane symmetrical to the other side. When the lugs enter the hooks’ grooves on the two symmetrical sides, the ladle is balanced by the symmetrical distribution of forces. To ensure reliable matching of the hook and the lug, it is necessary to ensure that the lug can enter the hook’s groove in the XOY plane on both sides. Therefore, the matching conditions between the hook and the lug can be reduced to the two-dimensional XOY plane, and the position of the lug and the hook along the direction of the *z*-axis per se has no impact on matching.

**Figure 3:**
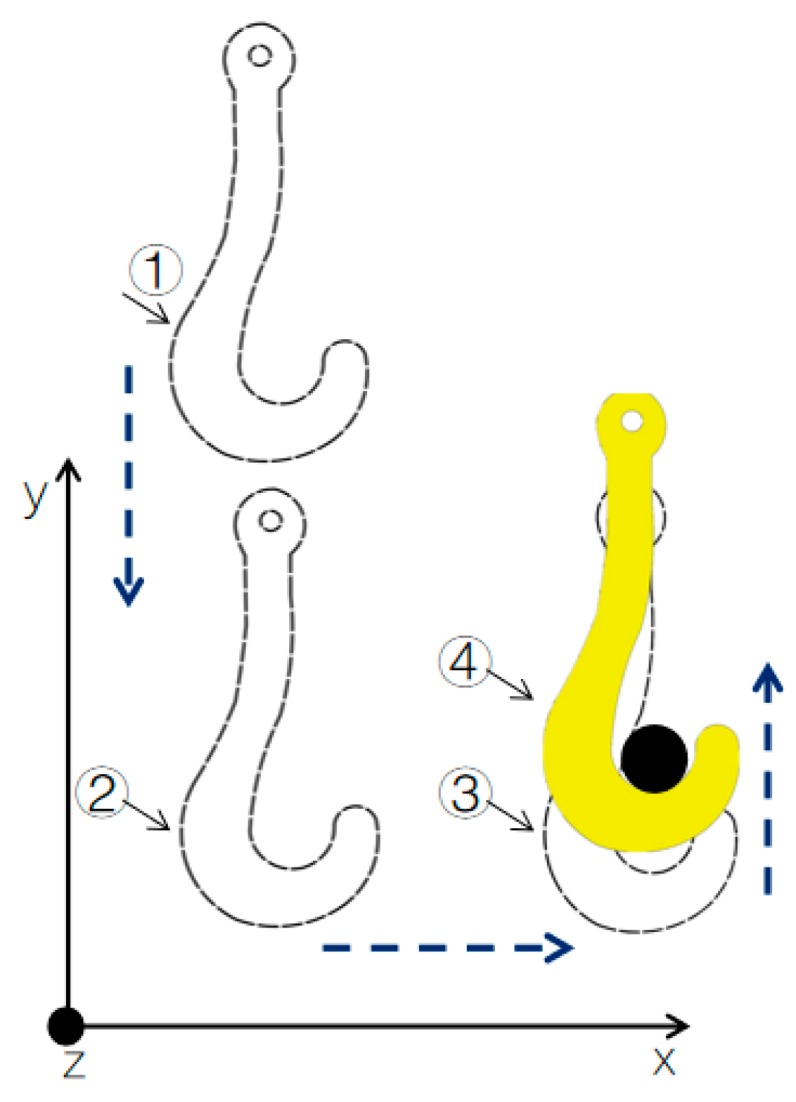
The matching process of the hook and the lug.

As shown in Figure 3, in the above XOY plane, the matching process of the hook and the lug is dissected in three steps:

(1) The hook moves from position ① to position ② along the direction of the *y*-axis, so the height of the hook’s groove is lower than that of the lug;

(2) The hook moves from position ② to position ③ along the direction of the *x*-axis, such that the hook groove is just under the lug;

(3) By lifting the hook along the direction of the *y*-axis from position ③ to position ④, the lug can enter the hook’s groove, which results in a reliable match between the lug and the hook.

**Figure 4:**
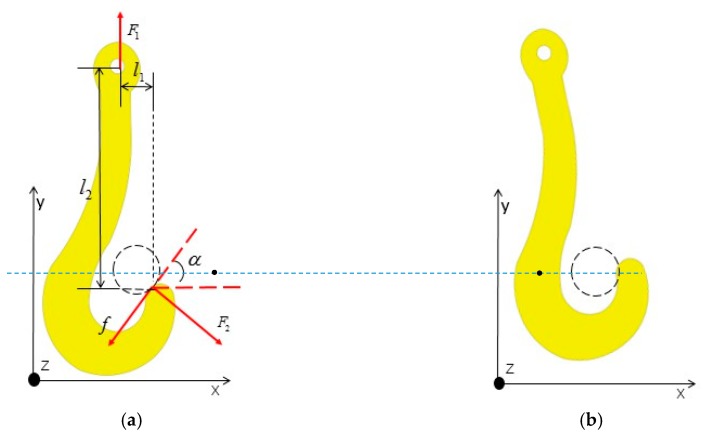
Hook rotation by force: (**a**) hook force analysis; (**b**) hook rotation.

In the above process, the key to ensuring reliable matching between the hook and the lug is the alignment of the hook’s groove and the lug along the direction of the *x*-axis in step (2) before lifting the hook. Along the direction of the *y*-axis, it is only required that the hook groove be lower than the lug. In actual production, the hook cannot be completely aligned with the lug, as there is always a position deviation between the two relative to the ideal situation. Under position deviation, the matching of the hook and the lug in step (3) has two statuses:

(1) Contact at the tip: As shown in Figure 4a, the lug is in contact with the hook’s circular arc tip. The ideal situation is that the lug can enter the hook’s groove within a certain extent of position deviation. To this end, the hook will rotate freely around its rotating axis when being lifted, as displayed in Figure 4b. The lug will remain stationary because the mass of the ladle is much larger than that of the hook. At this time, the lug can enter the hook’s groove via the composition of relative rotation and sliding.

The force analysis of the hook is demonstrated in Figure 4a: f is the friction force between the lug and the hook; $F_{1}$ is the traction force of the gantry crane to the lifting hook; $F_{2}$ is the elastic force between the lugs and the hook; and the angle between $F_{2}$ and the X axis is α. $l_{1}$ and $l_{2}$ are the distances from the contact point of the lug and the hook to the center of the hook’s rotating axis along the directions of the *x*- and *y*-axes, separately.

To ensure the rotation and sliding between the hook and the lug, the force of the hook should meet the following conditions.

(1) The static friction force $f_{0}$ between the lug and the hook is less than the projection of traction force $F_{1}$ along the direction of friction. The conditions ensuring relative sliding are

(1) $$\{\begin{array}{c} F_{1}sin\alpha >f_{0}\\ f_{0}=\mu_{0}F_{2}\\ F_{2}=F_{1}cos\alpha \end{array},$$

where $\mu_{0}$ is the static friction coefficient between the lug and the hook.

(2) In regard to rotation, the hook’s counterclockwise torque, relative to the center of the lug, is greater than its clockwise torque. As a result, the hook will rotate counterclockwise. The following equations should thus be satisfied:

(2) $$\{\begin{array}{c} \left(F_{2x}-f_{x}\right)l_{2}-\left(f_{y}+F_{2y}\right)l_{2}>0\\ F_{2x}=F_{2}cos\alpha \\ F_{2y}=F_{1}sin\alpha \\ f_{x}=fcos\alpha \\ f_{y}=fsin\alpha \\ f=\mu F_{2} \end{array},$$

where μ is the sliding friction coefficient between the lug and the hook.

When considering the size of a 75-ton ladle and the supporting gantry crane with strict position requirements as a reference, $\mu_{0}=0.7$ and μ=0.5 are selected for cast steel [15]. By substituting the relevant parameters into Equations (1) and (2), the relative position for the lug to enter the hook’s groove satisfies α > 35°.

At this point, the X coordinates of the lug’s centroid, $x_{1}$, and those of the hook groove’s centroid, $x_{2}$, meet:

(3) $$\delta x=\left|x_{1}-x_{2}\right|=\left(R_{1}+R_{2}\right)\left(1-sin\alpha \right).$$

According to the ladle’s design, it is known that *R*_1_ = 10 cm (the hook tip’s radius) and *R*_2_ = 14.5 cm (the lug’s radius). Under this condition, the maximum position deviation for reliable matching is $\delta_{x,max}$ = 10.47 cm.

(2) Contact at the long handle: The lug is in contact with the long handle of the hook. The force between the lug and the hook will cause the hook to rotate clockwise at a certain angle. While the hook is being lifted, the lifting hook has a tendency to rotate counterclockwise and restore its vertical orientation under gravity, so that the long handle of the hook is always in contact with the lug. Similar to status 1, rotation and sliding similar to what is presented in Figure 4b occur. From this, the lug finally enters the hook’s groove along the long handle. Under this condition, the lug can always enter the hook’s groove to achieve reliable matching. Therefore, the focus of this study is an analysis of the status “Contact at the tip”.

### 2.2. SAW RFID Localization Analysis

Combined with the analysis in the above section, before the hook is lifted in step (3), the relative position between the lug and the hook’s groove along the direction of the *x*-axis $\delta_{x}$ must satisfy $\delta_{x}$ < 10.47 cm. Thus, the problem of reliable matching is transformed into a problem of localization.

In a certain space area, the relative position of the lug and the hook are estimated through SAW RFID localization. After obtaining the *x*-coordinates of the lug and the hook’s groove $x_{1}$ and $x_{2}$, reliable matching between the hook and the lug can be determined:

(4) $$\{\begin{array}{c} \delta x>\Delta :\;Reliable\;Matching\\ \delta x<\Delta :Unreliable\;Matching, \end{array}$$

where Δ = 3 cm is the control margin according to the technical level of the gantry crane control [16], which is reserved for further adjustment in case of unreliable matching.

Therefore, the reliable matching detection requires that the localization error of relative position $\delta_{x}$ be no more than 7.4 cm.

## 3. Basis of SAW RFID Localization

### 3.1. SAW RFID System

**Figure 5:**
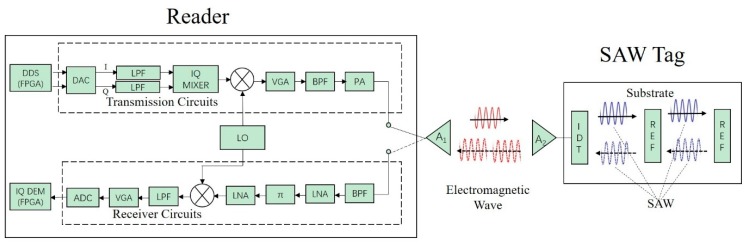
Basic architecture of the surface acoustic wave radio frequency identification (SAW) RFID system: DDS—direct digital synthesizer, DAC—digital to analog converter, LPF—low-pass filter, LO—local oscillator, VGA—variable gain amplifier, PA—power amplifier, A—antenna, π-π attenuator, LNA—low noise amplifier, ADC—analog to digital converter, IQ DEM—IQ demodulator, IDT—interdigital transducer, and REF—reflector.

In this paper, the SAW RFID system works with a fixed-frequency time-domain pulse, and its frequency bandwidth ranges from 915 MHz to 930 MHz. The basic architecture of the SAW RFID system is shown in Figure 5. This system is composed of a SAW RFID reader and a SAW tag with delay-line structure.

The reader is composed of transmission circuits, receiver circuits, a field-programmable gate array (FPGA), and an antenna. The reader antenna is connected to the transmission and receiver circuits via a high-speed switch for transmitter–receiver isolation. FPGA, the core of the reader, is responsible for reader configuration, signal transmission, and signal processing. For the sake of simplicity, connections between FPGA and the circuits are omitted in Figure 5.

The SAW tag is composed of a piezoelectric substrate, an interdigital transducer (IDT), reflectors, and an antenna. The IDT is connected to the antenna and lies on one side of the substrate. Along the substrate, there exist multiple reflectors in different positions.

When the reader interrogates the SAW tag, the working process of the SAW RFID system is:

(1) The FPGA enables transmission circuits in the upper dashed box, and the direct digital synthesizer (DDS) provides in-phase/quadrature (IQ) digital pulses, which are converted into analog pulses by a digital-to-analog converter (DAC) and are IQ modulated by an IQ mixer. Then, the modulated signal is mixed with a local oscillator (LO) signal to generate the radio frequency (RF) signal. After that, the RF pulse signal is amplified by a series of amplifiers and sent to the reader antenna. After transmission of RF electromagnetic (EM) pulse, FPGA disables the transmission circuits.

(2) The EM pulse signal is received by the SAW tag’s antenna. Then, the IDT, which is connected to the antenna, converts the EM wave into SAW via an inverse piezoelectric effect. The SAW pulse has the same frequency as the EM pulse and propagates on the tag’s substrate. After a few microseconds of propagation on the tag’s piezoelectric substrate, part of the SAW is reflected by the reflectors and returns to the IDT. Then, the SAW is converted into an EM wave via a piezoelectric effect and sent to the tag’s antenna.

(3) A few microseconds after the signal’s transmission, the FPGA enables receiver circuits in the lower dashed box. After multi-stage amplification and filtering, the received signal is mixed with the LO signal to obtain the base band signal. After being sampled by the analog-to-digital converter (ADC), the digital signal is sent to FPGA, after which the receiver circuits are disabled. Finally, the FPGA employs IQ demodulation to obtain signal features like time-delay, signal strength, and signal phase, and the interrogation is finished.

Due to the propagation of SAW (at a speed of around 3000 m/s, depending on the piezoelectric substrate) in the delay-line SAW tag, the reader will receive the tag’s backscattered signal after a few microseconds. Between the tag signal’s transmission and reception, unexpected signals, which are not backscattered by the tag, will propagate in the space at the speed of light and be attenuated greatly. Therefore, the unexpected signal is suppressed by the tag’s time delay and has no impact on the tag’s backscattered signal.

### 3.2. Basic Principle of Phase Based Localization

The features of the SAW tag’s backscattered signal, such as time-delay, signal strength, and signal phase, are the basis of SAW RFID localization. With the spatial propagation of the EM signal, these features change with different distances between the tag and the reader antenna. Therefore, if the location of the reader antenna is fixed, the signal features will change with different tag positions, which means that the tag’s position can be inferred from the signal features.

In terms of localization accuracy, the measurement of signal strength can be easily affected by the environment and, therefore, is not stable. Furthermore, the measurement precision of the time-delay is limited by the SAW tag’s bandwidth [8], which means that both features cannot be applied to SAW RFID localization with the desired accuracy.

In contrast, phase-based SAW RFID localization is suitable for matching detection. After IQ demodulation, the backscattered phase φ is obtained:

(5) $$\varphi =\left(\varphi_{d}+\phi \right)mod2\pi ,$$

among which

(6) $$\{\begin{array}{c} \varphi_{d}=2\pi \frac{2d}{\lambda }=4\pi \frac{fd}{c}\\ \phi =\phi_{Tx}+\phi_{Rx}+\phi_{Tag}+\pi \end{array},$$

where d is the propagation distance from the tag to the reader antenna, λ and f are the wavelength and frequency of the EM pulse, and c is the speed of light. Furthermore, $\phi_{Tx}$ and $\phi_{Rx}$ are the phase offsets caused by the transmission and receiver circuits, respectively. In addition, $\phi_{Tag}$ is the phase offset caused by SAW propagation on the tag, and π is the phase offset caused by the SAW tag reflectors.

When distance d changes Δd = 1 mm due to the tag’s displacement, phase change $\Delta \varphi_{d}=4\pi \frac{f\Delta d}{c}$ equals 2.19–2.23° for the EM wave with frequency 915–930 MHz. Generally, it is easy to achieve a degree level of resolution for the backscattered phase, which corresponds to the *mm* level resolution of the tag’s displacement. Combined with the fixed position of the reader antenna, the tag’s position can be estimated with millimeter-level high precision.

### 3.3. Phase for SAW RFID Localization

In SAW RFID localization, only phase $\varphi_{d}$ caused by spatial propagation is involved; the phase offsets caused by other factors should be compensated.

For the phase offsets $\phi_{Tx}$ and $\phi_{Rx}$ from the reader circuits, the receiver circuits can be enabled earlier to capture the leakage signal. The leakage signal passes directly from the transmission circuits to the receiver circuits in signal transmission. Therefore, its phase equals $\phi_{Tx}+\phi_{Rx}$, with which the reader’s phase offset can be compensated.

SAW propagation in the SAW tag result in the phase offset, $\phi_{Tag}$, which will be affected by environmental temperature. Phase offset $\phi_{Tag}$ provides a clear rule with temperature when the design of the SAW tag is completed [17], and the temperature and phase $\phi_{Tag}$ can be precisely measured and compensated by setting multiple reflectors in the SAW tag.

## 4. SAW RFID Localization for the Hook and Lug

Because of the 2π ambiguity in the backscattered phase φ, the unwrapped phase $\varphi_{d}$ and distance d cannot be computed directly. In this section, the localization methods based on geometric mapping (for the lug) and synthetic aperture (for the hook) are proposed, with backscattered phases towards the high-precision localization of the lug and the hook.

### 4.1. Setup of the SAW Tag and Reader Antenna

**Figure 6:**
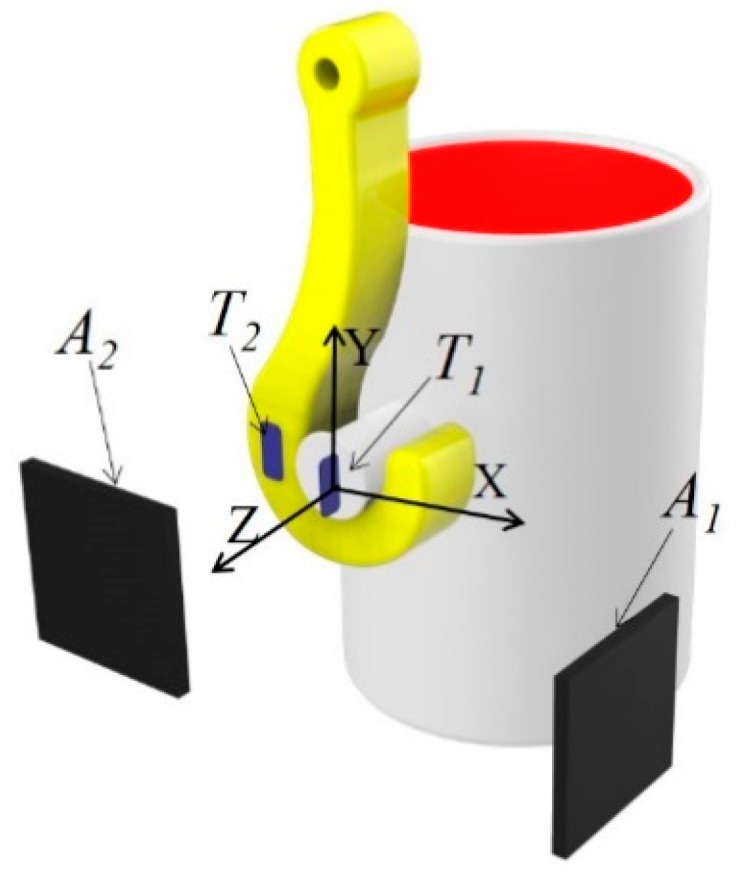
Setup of the SAW tag and reader antenna.

In this paper, two SAW tags, $T_{1},\;T_{2}$, and two reader antennas, $A_{1},\;A_{2}$, are used for localization of the hook and lug. As illustrated in Figure 6, in the center of the lug, tag $T_{1}$ is installed toward the positive direction of the *x*-axis, and reader antenna $A_{1}$, facing $T_{1}$, is arranged along the positive direction of the *x*-axis in the same XOY plane. Tag $T_{2}$, toward the positive direction of the *z*-axis, is installed on the long handle of the hook, and reader antenna $A_{2}$, facing $T_{2}$, is arranged along the positive direction of the *z*-axis.

The centroid coordinates of the above tags and antennas are deployed based on their spatial coordinates. The coordinates of tags $T_{1}$ and $T_{2}$ are denoted as $S_{T_{1,0}}=\left(x_{T_{1,0}},y_{T_{1,0,}},z_{T_{1,0,}}\right)$ and $S_{T_{2,0}}=\left(x_{T_{2,0}},y_{T_{2,0,}},z_{T_{2,0,}}\right)$, respectively, and the coordinates for antennas $A_{1}$ and $A_{2}$ are $S_{A_{1}}=\left(x_{A_{1}},y_{A_{1}},z_{A_{1}}\right)$ and $S_{A_{2}}=\left(x_{A_{2}},y_{A_{2}},z_{A_{2}}\right)$, respectively. During tag and antenna installation, it is required that $z_{T_{1,0}}=z_{A_{1}}$ and $y_{T_{2,0}}=y_{A_{2}}$.

Since the relative position between tag $T_{1}$ and the lug, as well as tag $T_{2}$ and the hook, are known, the relative position of the lug and the hook are also known, provided that the relative position between tags $T_{1}$ and $T_{2}$ is obtained.

### 4.2. Lug Localization Method Based on Geometric Mapping

Let the real-time coordinate of $T_{1}$ be $S_{T_{1}}=(x_{T_{1}},y_{T_{1}},z_{T_{1}})$. Due to the ladle’s position deviation, the tag’s coordinate $S_{T_{1}}$ has a deviation of $\Delta x_{1}$ and $\Delta z_{1}$ along the direction of the *x*- and *z*-axes, respectively, relative to its initial coordinate, $S_{T_{1,0}}$. Given that the lug is fixed on the ladle, deviation along the *y*-axis $\Delta y_{1}=0$. In this section, by combining the prior information of the lug’s position and a special arrangement of the tag and reader antenna, deviation $\Delta x_{1}$ is mapped onto distance change Δd between tag $T_{1}$ and reader antenna $A_{1}$, where Δd is no more than half a wavelength. Therefore, Δd is obtained from the backscattered phase, and then $x_{T_{1}}$ is estimated to achieve localization of the lug.

The real-time distance d from tag $T_{1}$ to antenna $A_{1}$ is

(7) $$d=\sqrt{{\left(x_{T_{1}}-x_{A_{1}}\right)}^{2}+{\left(y_{T_{1}}-y_{A_{1}}\right)}^{2}+{\left(z_{T_{1}}-z_{1}\right)}^{2}}.$$

According to the Taylor expansion of d using the coordinates $S_{T_{1,0}}$, the linear term $\Delta x_{1}$ and $\Delta z_{1}$ are retained:

(8) $$d\approx d_{0}+\Delta x_{1}\frac{\left(x_{T_{1,0}}-x_{A_{1}}\right)}{d_{0}},$$

where $d_{0}$ is the distance from the tag coordinates, and $S_{T_{1,0}}$ to the antenna coordinates $S_{A_{1}}$.

By adjusting antenna $A_{1}$‘s height, $y_{A_{1}}$, $d_{0}$ satisfies

(9) $$d_{0}=m*\left(x_{T_{1,0}}-x_{A_{1}}\right).$$

Substituting Equation (9) into Equation (8), we can get

(10) $$\Delta d=d_{1}-d_{0}=\frac{\Delta x_{1}}{m}.$$

The above geometric approximation in Equations (8)–(10) maps m half-wavelength $\Delta x_{1}$ into the distance change, Δd, within one half-wavelength by antenna arrangement. Since Δd is no more than one half-wavelength, Δd can be estimated unambiguously by measuring the backscattered phase $\varphi_{T_{1,0}}$ at $S_{T_{1,0}}$ and $\varphi_{T_{1}}$ of $S_{T_{1}}$:

(11) $$\Delta d=\frac{\lambda }{4\pi }\left(\varphi_{T_{1}}-\varphi_{T_{1,0}}\right).$$

As a result, $x_{T_{1}}$ can be determined:

(12) $$x_{T_{1}}=x_{T_{1,0}}+\Delta x_{1}=x_{T_{1,0}}+m\Delta d.$$

In this section, the tag’s X coordinate $x_{T_{1}}$ and the lug’s coordinate $x_{1}$ are obtained by geometric mapping and phase-based ranging using a special arrangement of the tag and reader antenna.

### 4.3. Hook Localization Method Based on Synthetic Aperture

The previous section has discussed the localization of the lug fixed on the steel ladle, whose position satisfies Δy=0. However, for the hook’s localization, the hook’s trajectory has deviations along the direction of the *x*, *y*, and *z*-axes. In this regard, $\Delta y_{2}\neq 0$ violates the geometric mapping requirement in Equations (7)–(9). Therefore, this study proposes considering the prior knowledge of the hook’s movement pattern in matching step (2), as mentioned in Section 2.1. Consequently, a phase-based positioning method with synthetic aperture is introduced to achieve localization of the hook. The details are as follows.

**Figure 7:**
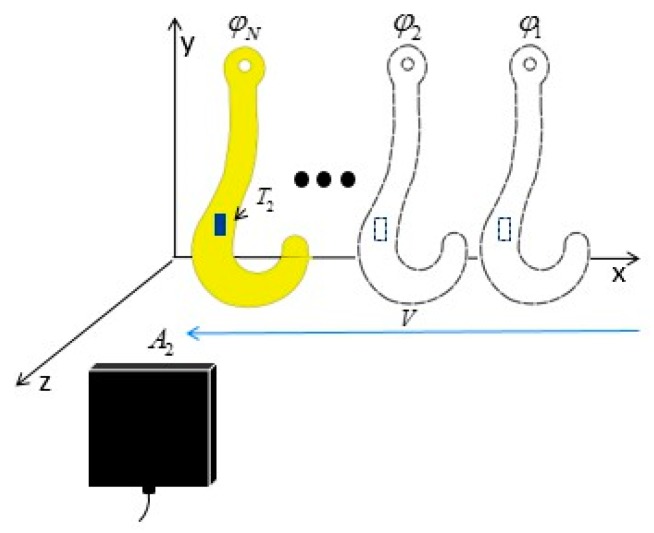
The reader interrogates the moving tag.

As illustrated in Figure 7, tag $T_{2}$ moves with the hook along the direction of the *x*-axis, and the speed is approximated to be constant. Let the speed be $V=\left(v_{x},0,0\right)$. Because the reader keeps interrogating the tag from antenna $A_{2}$ at a fixed time interval, IRT, a phase vector of length N is obtained, denoted as $\vec{\varphi }=\left[\varphi_{1},\varphi_{2},\ldots \varphi_{N}\right]$. Additionally, tag $T_{2}$‘s coordinates at $\varphi_{0}$ are denoted as $S_{T_{2,1}}=\left(x_{T_{2,1}},y_{T_{2,1}},z_{T_{2,1}}\right)$.

**Figure 8:**
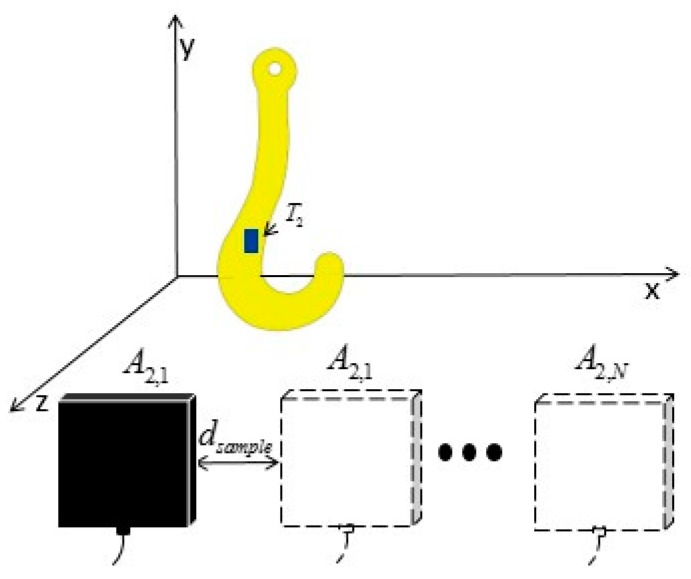
Virtual antenna array.

From the angle of relative motion, tag $T_{2}$ is considered to be stationary with respect to the hook. The reader antenna $A_{2}$ moves from $S_{A_{2}}$ at the speed of −V and reads the tag at different positions to obtain $\vec{\varphi }$. Then, the propagation time of the electromagnetic wave (ns magnitude) and the propagation time of SAW (us magnitude) can be properly ignored in comparison to the sampling interval IRT, which is at the ms level. As a result, antenna $A_{2}$ can be considered to interrogate tag $T_{2}$ at equal space intervals along the direction of the *x*-axis. This is equivalent to a virtual antenna array $A_{2,1},A_{2,2},\ldots A_{2,N}$ interrogating tag $T_{2}$ located at the $S_{T_{2,1}}$, as shown in Figure 8, thus resembling a synthetic aperture radar (SAR). We can estimate the position of tag $T_{2}$ using the synthetic aperture technique.

The coordinate $S_{2,n}$ of array element $A_{2,n}$ is

(13) $$S_{A_{2,n}}=S_{A_{2}}-n*IRT*V,n=1,2,\ldots N.$$

Phase $\varphi_{n}$, read by the antenna element $A_{2,n}$, can be expressed as

(14) $$\varphi_{n}=\frac{4\pi d_{n}}{\lambda }\mathrm{mod}2\pi ,n=1,2,\ldots N,$$

where $d_{n}$ is the distance from array element $A_{2,n}$ to tag $T_{2}$:

(15) $$d_{n}=\left|S_{A_{2,n}}-S_{T_{2,1}}\right|.$$

In a hoisting operation, $y_{T_{2,1}}$, $z_{T_{2,1}}$, $y_{A_{2}}$, and $z_{A_{2}}$ are constant values; the ideal value of $\varphi_{n}$ reaches an extreme value at $x_{A,n}=x_{T_{2,1}}$, and $\vec{\varphi }$ is symmetrical around this position. Since the position of the antenna element is known, $\vec{\varphi }$ distribution is determined only by $x_{T_{2,1}}$. Deviation $\Delta y_{T_{2,1}}$ and $\Delta z_{T_{2,1}}$ of $y_{T_{2,1}}$ and $z_{T_{2,1}}$ cause an overall offset only in this phase distribution. Therefore, $\vec{\varphi }$ is controlled by $x_{T_{2,1}}$, and $x_{T_{2,1}}$ can be obtained based on the following relationship:

(16) $$x_{T_{2}}=x_{T_{2,1}}+\left(N-1\right)IRT*v_{x}.$$

According to the antenna element’s coordinates, $S_{2,n}$, a set of backscattered phases, $\vec{\theta }=\left[\theta_{1},\theta_{2},\ldots \theta_{N}\right],$ related only to the X coordinates, $x_{T_{2}}^{\prime }$, of the tag is generated according to Equations (13)–(15):

(17) $$\theta_{n}=\frac{4\pi }{\lambda }\left|S_{A_{2,n}}-S_{0}^{\prime }\right|\mathrm{mod}2\pi .$$

Among these, $S_{0}^{\prime }=\left(x_{T_{2}}^{\prime },y_{T_{2}}^{\prime },z_{T_{2}}^{\prime }\right)$ may use the *y*- and *z*-coordinates as the initial values, $y_{T_{2}}^{\prime }=y_{T_{2,0}}$ and $z_{T_{2}}^{\prime }=z_{T_{2,0}}$.

Similar to $\vec{\varphi }$, $\vec{\theta }$ also presents the same regularity around $x_{T_{2}}^{\prime }$. When $x_{T_{2}}^{\prime }=x_{T_{2,1}}$, $\vec{\theta }$ is closest to $\vec{\varphi }$. For a certain range of $x_{T_{2}}^{\prime }$, $\vec{\theta }$ and $\vec{\varphi }$ are pattern matched; then, the $x_{T_{2}}^{\prime }$ that best matches $\vec{\theta }$ and $\vec{\varphi }$ is the best estimate ${\hat{x}}_{T_{2,1}}$ of $x_{T_{2,1}}$ [18].

For pattern matching, $\vec{\varphi }$ and $\vec{\theta }$ are normalized:

(18) $$\{\begin{array}{c} {\vec{\varphi }}_{nom}=[\exp\left(-j\varphi_{1}\right),\exp\left(-j\varphi_{2}\right),\ldots .\exp\left(-j\varphi_{N}\right)]\\ {\vec{\theta }}_{nom}=[\exp\left(-j\theta_{1}\right),\exp\left(-j\theta_{2}\right),\ldots .\exp\left(-j\theta_{N}\right)] \end{array}.$$

Then, $x_{T_{2,1}}$’s best estimation of ${\hat{x}}_{T_{2,1}}$ is [19]

(19) $${\hat{x}}_{T_{2,1}}=\arg max\frac{\left|{\vec{\theta }}_{nom}{\vec{\varphi }}_{nom}\right|}{{\left\|{\vec{\theta }}_{nom}\right\|}^{2}{\left\|{\vec{\varphi }}_{nom}\right\|}^{2}},$$

where H is the Hermitian operator, and ‖ ‖ is the norm.

In this section, combined with prior knowledge of the hook’s movement, the tag’s *x*-coordinate $x_{T_{2}}$ is obtained by the synthetic aperture technique. In this way, the hook’s *x*-coordinate $x_{2}$ is obtained.

### 4.4. Relative Localization of the Hook and Lug

**Figure 9:**
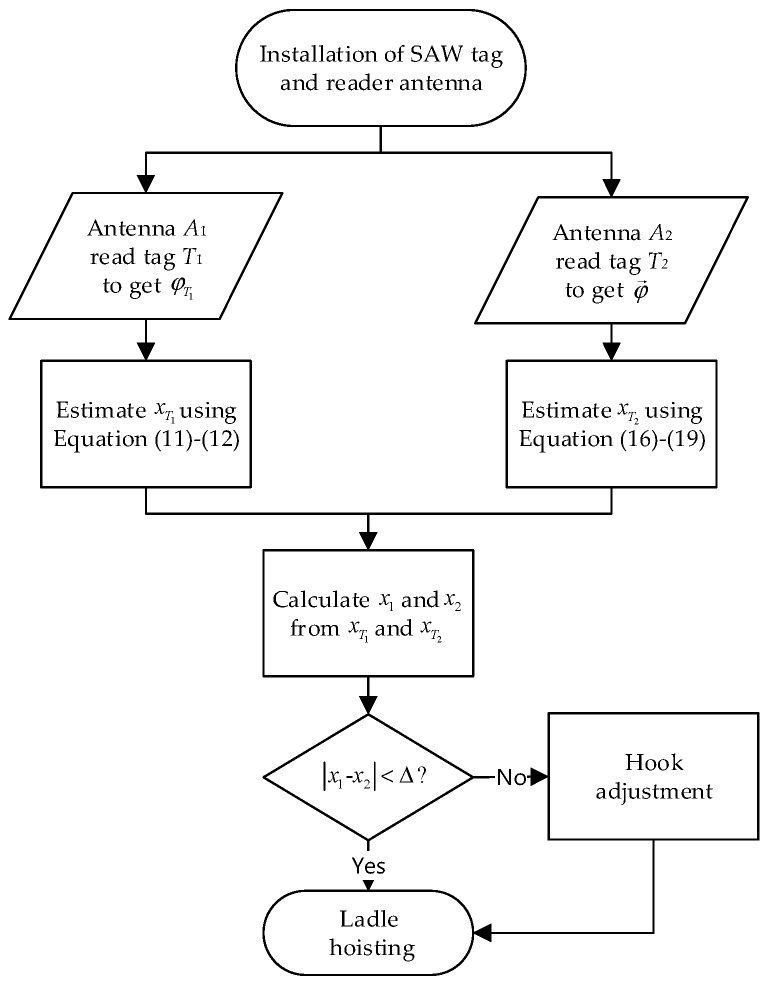
Diagram of the reliable matching detection.

In Section 3.1 and Section 3.2, we have introduced localization of the lug based on geometric mapping and localization of the hook based on synthetic aperture for the purpose of matching detection. To summarize this work, the reliable matching detection methods are summarized in Figure 9.

## 5. Numerical Simulations

To verify the feasibility of the SAW RFID localization method, the simulation experiments have been carried out using the phases calculated in Equation (5). The parameters used in the simulation in this section are set according to the actual scene. The wavelength within the SAW tag’s bandwidth is similar; therefore, the interrogation frequency f = 922.5 MHz is selected without a loss of generality. By carrying out multiple phase measurement experiments, the phase errors between the measurement values and theoretical values in Equation (5) have been evaluated, and the errors satisfy δφ < 5°. The space coordinates of the lug and the hook are set according to the actual size in the steelmaking workshop.

### 5.1. Lug Localization Simulation

Tag $T_{1}$’s coordinates are set as $S_{T_{1,0}}=\left(0,5,0\right)$ and the antenna $A_{1}$’s coordinates are $S_{A_{1,0}}=\left(2,1.5,0\right)$. According to the actual scene, the tag’s position deviation satisfies −15 cm < $\left[\Delta x_{1},\Delta z_{1}\right]$ < 15 cm.

**Figure 10:**
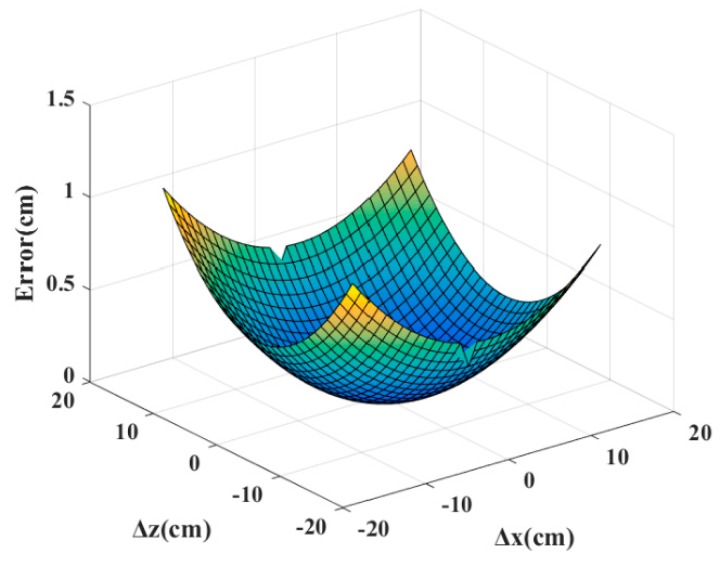
Localization error of the geometric mapping method.

Since geometric mapping includes numerical approximation, the positioning accuracy of the geometric mapping is determined under ideal conditions without phase error. The location errors under the different deviations, $\Delta x_{1}$ and $\Delta z_{1}$, are simulated in steps of 1 cm. The results of the numerical simulation are illustrated in Figure 10. It can be seen that the theoretical maximum location error of the geometric mapping is only 1.13 cm.

The geometric mapping method maps the deviation of several half wavelengths along the direction of the *x*-axis into a distance change within one half wavelength, which reduces the sensitivity of the phase to distance and eliminates 2π ambiguity. However, the influence of the phase error on location accuracy is enhanced, so the influence of the phase error should be considered.

**Figure 11:**
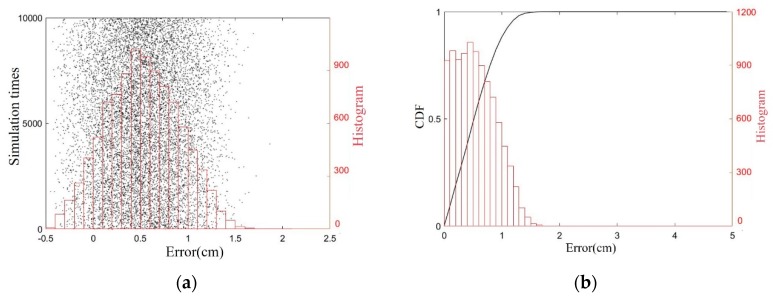
Simulation results of lug localization. (**a**) location errors and its histogram; (**b**) the cumulative distribution function (CDF) and histogram of the errors’ absolute values.

A phase error of δφ < 5° has been added to the above simulation, and a 10^4^ times value simulation has been performed, and location errors of $x_{1}$ are displayed in Figure 11. The results shows that positive errors have a larger range that the negative ones because positive $\Delta x_{1}$ will reduce distance d and enlarge the approximation error. Meanwhile, statistical histogram and cumulative distribution function (CDF) have been used to illustrate the distribution of the errors in Figure 11, which show that the location error of the lug’s *x*-coordinate is no greater than 1.7 cm, with a probability of 99.99%, a maximum error of 1.92 cm, and a root mean square error of 0.46 cm.

### 5.2. Hook Localization Simulation

For this simulation, the following parameters were set: tag $T_{2}$’s coordinates are $S_{T_{2,0}}=\left(2,4.5,0\right)$; antenna $A_{2}$’s coordinates are $S_{A_{2}}=\left(1,4.5,2\right)$; the hook’s speed is V=(−0.2,0,0); the reader’s interrogation interval IRT = 50 ms; the interrogate number is N=200; the phase measurement error is δφ < 5°; and the $x_{T_{2}}^{\prime }$ scanning step is 0.5 cm.

**Figure 12:**
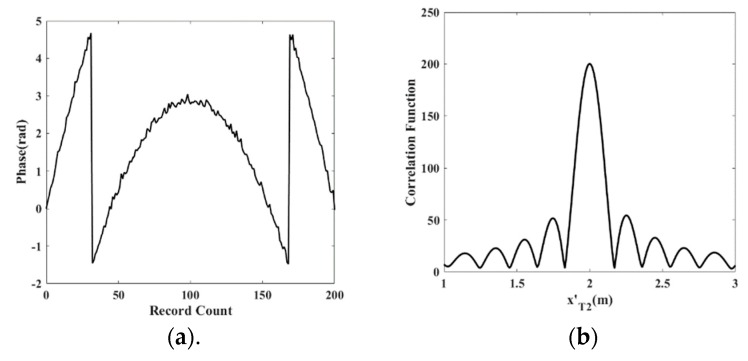
Illustration of the synthetic aperture method. (**a**) phase record $\vec{\varphi }$ in one simulation; (**b**) correlation functions versus $x_{T_{2}}^{\prime }$.

Since the phase error is random, the phase error does not affect the distribution law of $\vec{\varphi }$ and, therefore, does not affect the location accuracy, which is verified by 10^4^ digital simulations with phase error. Phase $\vec{\varphi }$ and the value of the correlation function of ${\vec{\varphi }}_{nom}$ and ${\vec{\theta }}_{nom}$ in one simulation experiment are shown in Figure 12. The correlation function reaches a maximum value at $x_{T_{2}}^{\prime }$ = 2.01 m.

**Figure 13:**
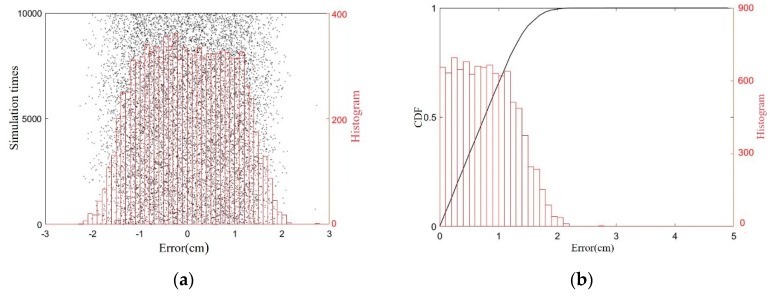
Simulation results of hook localization. (**a**) location errors and its histogram; (**b**) the CDF and histogram of the errors’ absolute values.

Afterwards, the effect of the position deviation is considered. Similar to Section 5.1, the position deviation satisfies −15 cm < $\left[\Delta x_{2},\Delta y_{2},\Delta z_{2}\right]$ < 15 cm, and a 10^4^ times value simulation has been performed. The location errors of $x_{2}$ as well as error histogram and CDF were displayed in Figure 13. The simulations show that the error of the *x*-coordinate estimation of the synthetic aperture method does not exceed 2.61 cm with a probability of 99.99%, a maximum error of 2.72 cm, and a root mean square error of 0.61 cm.

### 5.3. Relative Localization Simulation

**Figure 14:**
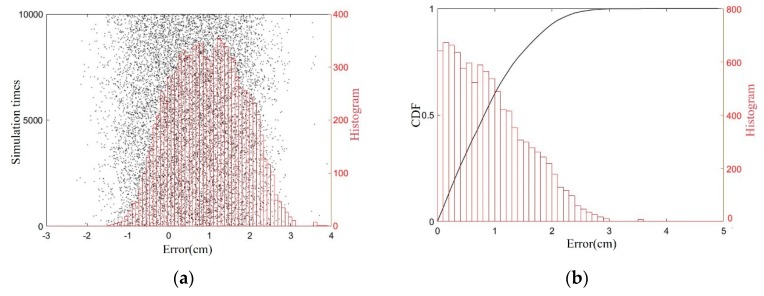
Simulation results of relative localization. (**a**) location errors and its histogram; (**b**) the CDF and histogram of the errors’ absolute values.

According to the above parameter settings, 10^4^ times simulations have been carried out to verify the relative localization accuracy of the hook and lug—namely, the error of the estimated relative position $\left|x_{1}-x_{2}\right|$. Similarly, the combined errors and statistic results are provided in Figure 14. The location error is less than 3.71 cm, with a probability of 99.99%, a maximum error of 3.93 cm, and a root mean square error of 0.82 cm. According to the analysis in Section 2.2, the location accuracy should be less than 7.4 cm to realize the matching detection. Therefore, the accuracy of the proposed method meets the requirements of reliable matching detection.

## 6. Experimental Results

### 6.1. Configuration of the SAW RFID System

**Figure 15:**
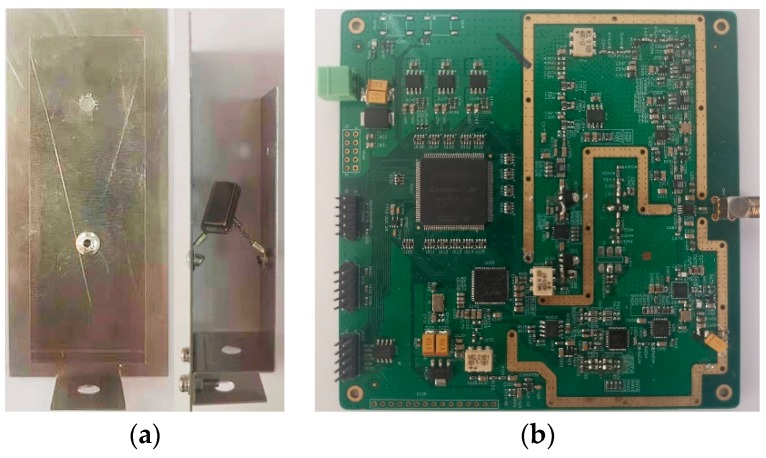
The self-developed SAW RFID system, (**a**) SAW tag and (**b**) reader circuits.

In this work, the SAW tag is designed as follows for the localization of the hook and lug, as displayed in Figure 15. (1) To ensure the reading distance of the SAW tag and reduce the transmission power required, 128°YX-cut LiNbO_3_ is used as the tag’s substrate, and a secondary harmonic single-phase unidirectional transducer (SPUDT) is designed [20]. (2) Each SAW tag uses multiple reflectors for temperature measurement and compensation. Meanwhile, according to the reading test in the steelmaking workshop, the distance between the first reflector and the transducer is greater than 3.484 mm to suppress environmental clutter. (3) In order to distinguish the backscattered signals of each tag, the two SAW tags are separated by the time domain [21,22]. (4) Meanwhile, the tag is equipped with a directional antenna. The antenna has a gain of 2.5 dBi and a half power beamwidth (HPBW) of 54° and 60° on the horizontal and vertical planes (Simulated data in ANSYS HFSS (ANSYS, Pittsburgh, PA, USA)). The directional antenna with a narrower beamwidth is able to prevent the tag from environmentally reflecting signals coming from unexpected angles [23].

The configuration parameters of the self-developed SAW reader are given in Table 1, and the reader circuit is shown in Figure 15.

### 6.2. Physical Verification Experiment

To further verify the feasibility of the proposed SAW RFID localization method under real-world conditions, the self-developed SAW RFID system has been used for physical verification experiments. In the real test, the reader and SAW tag can work reliably within 5 m, which meets the proposed method’s requirements for tag interrogation.

**Figure 16:**
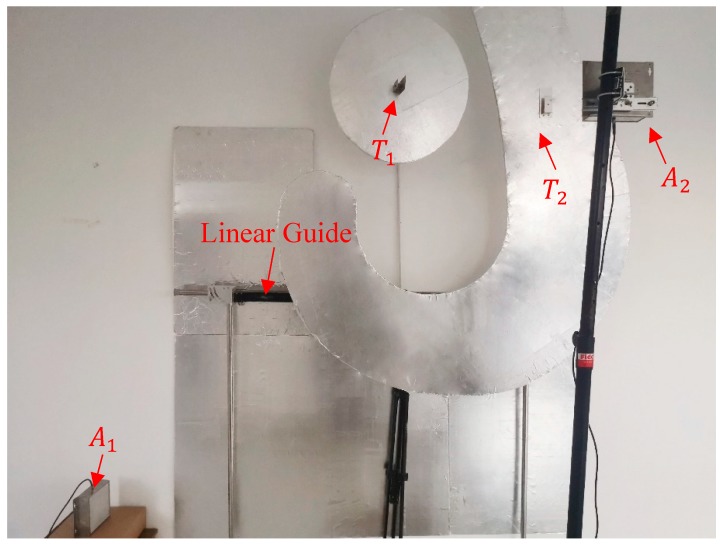
Experimental scene.

The experimental platform shown in Figure 16 has been built to simulate the actual matching scene of the hook and the lug. Aluminum foil has been used to simulate the metallization environment in a steel-making workshop, and a linear guide has been used to replace the gantry crane to drive the hook’s movement. Due to the limitations of the lab environment, the arrangement of the tags and reader antennas satisfies

(20) $$\{\begin{array}{c} S_{T_{1,0}}=\left(0,2,0\right)\\ S_{T_{2,0}}=\left(1,2,0\right)\\ S_{A_{1}}=\left(-1,0.25,0\right)\\ S_{A_{2}}=\left(0.5,2,1.2\right) \end{array}.$$

Despite the differences in the experimental settings, the platform is believed to be able to verify the feasibility of the proposed method to some extent. (1) For an ultra-high frequency (UHF) EM wave, aluminum has an even stronger reflection ability than cast steel [24]; therefore, it is reasonable to replace cast steel with aluminum. (2) Meanwhile, for the SAW RFID localization method in Section 4, a reduction in the experimental size will enlarge the approximation error and blur the relationship between $\vec{\varphi }$ and $x_{T_{2,1}}$’, which will result in a larger localization error. Therefore, laboratory experiments with a smaller size are able to prove this method’s localization accuracy.

**Figure 17:**
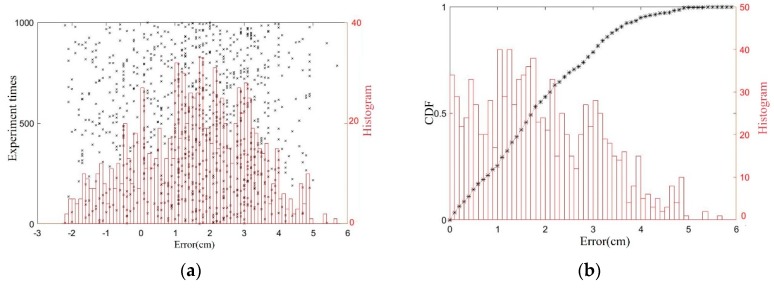
Experimental results. (**a**) location errors and its histogram; (**b**) the CDF and histogram of the errors’ absolute values.

Within a range of −15 cm < $\left[\Delta x_{1},\Delta z_{1},\Delta x_{2},\Delta y_{2},\Delta z_{2}\right]$ < 15 cm position deviations, the position of the lug and trajectory of the hook are randomly selected, and the location errors of $\left|x_{1}-x_{2}\right|$ has been obtained by conducting 1000 repeated localization experiments. The location errors and its static results process are presented in Figure 17. The relative position error is less than 4 cm with a probability of 96.2%. The maximum error is 5.7 cm, and the root mean square error is 2.16 cm. The errors are larger than that in simulations, which is consistent with the analysis of experiments with small size. Meanwhile, the experimental results have shown that accuracy of the proposed method meets the requirements of reliable matching detection.

## 7. Conclusions

This paper proposes a method for reliable matching detection of the crane hook and ladle lug during ladle hoisting by using SAW RFID localization. Combined with a special arrangement of the tag and reader antenna, the position of the lug is estimated using geometric mapping and phase based ranging. Combined with prior knowledge of the hook’s movement, the position of the hook is estimated through the synthetic aperture technique. Numerical simulations and real-world experiments on a simulated platform have been carried out to verify the accuracy of the proposed localization method, thereby demonstrating the feasibility of reliable matching detection. However, a real environment in a steelmaking workshop may be more complex, and the application of this method still demands further research.

## Figures and Tables

**Table 1:** Configuration parameters of the reader.

Reader Transmission Power	28 dBm
Receiver sensitivity	−90 dBm
Working Frequency	915~930 MHz
Antenna Gain	9 dBi
Antenna Horizontal HPBW	68°
Antenna Vertical HPBW	60°
